# Structural Health Monitoring Impact Classification Method Based on Bayesian Neural Network

**DOI:** 10.3390/polym14193947

**Published:** 2022-09-21

**Authors:** Haofan Yu, Aldyandra Hami Seno, Zahra Sharif Khodaei, M. H. Ferri Aliabadi

**Affiliations:** Structural Integrity and Health Monitoring Group, Department of Aeronautics, Imperial College London, London SW7 2AZ, UK

**Keywords:** structural health monitoring, passive sensing, impact classification, Bayesian neural network, artificial neural network, uncertainty measurement

## Abstract

This paper proposes a novel method for multi-class classification and uncertainty quantification of impact events on a flat composite plate with a structural health monitoring (SHM) system by using a Bayesian neural network (BNN). Most of the existing research in passive sensing has focused on deterministic approaches for impact detection and characterization. However, there are variability in impact location, angle and energy in real operational conditions which results in uncertainty in the diagnosis. Therefore, this paper proposes a reliability-based impact characterization method based on BNN for the first time. Impact data are acquired by a passive sensing system of piezoelectric (PZT) sensors. Features extracted from the sensor signals, such as their transferred energy, frequency at maximum amplitude and time interval of the largest peak, are used to develop a BNN for impact classification (i.e., energy level). To test the robustness and reliability of the proposed model to impact variability, it is trained with perpendicular impacts and tested by variable angle impacts. The same dataset is further applied in a method called multi-artificial neural network (multi-ANN) to compare its ability in uncertainty quantification and its computational efficiency against the BNN for validation of the developed meta-model. It is demonstrated that both the BNN and multi-ANN can measure the uncertainty and confidence of the diagnosis from the prediction results. Both have very high performance in classifying impact energies when the networks are trained and tested with perpendicular impacts of different energy and location, with 94% and 98% reliable predictions for BNN and multi-ANN, respectively. However, both metamodels struggled to detect new impact scenarios (angled impacts) when the data set was not used in the development stage and only used for testing. Including additional features improved the performance of the networks in regularization; however, not to the acceptable accuracy. The BNN significantly outperforms the multi-ANN in computational time and resources. For perpendicular impacts, both methods can reach a reliable accuracy, while for angled impacts, the accuracy decreases but the uncertainty provides additional information that can be further used to improve the classification.

## 1. Introduction

The application of composite materials in the aerospace industry has grown in recent years owing to their unique properties. However, their mechanical properties can be significantly influenced by the occurrence of impact induced damages in composite, such as delamination and matrix cracking, which are difficult to identify [1,2,3]. Impact damage, from the perspective of detectability, can be classified as clearly visible impact damage (CVID) and barely visible impact damage (BVID). CVID can easily be detected in the process of regular maintenance while BVID is much more difficult to be detected and can be potentially hazardous to the safety of components or even the whole system. Non-destructive inspection (NDI) techniques, such as ultrasonic C-scan detection and acoustic emission (AE), are commonly used to check the integrity of composite and detect BVID [4,5]. However, the current NDI methods are time-consuming, expensive and require access to the part. Another challenge of NDI is the lack of continuous monitoring [6]. Therefore, a more convenient, cheaper and faster method is urgently needed for BVID detection. Structural health monitoring (SHM) has become a popular method for BVID detection as it reduces the cost of detection and maintenance, and most importantly, achieves continuous monitoring by using a network of sensors [7]. The real-time data collected from sensors contain the knowledge of the real behavior of the structure in service. By analyzing these data, the energy of an external impact can be extrapolated, and the influence of damage can be assessed to determine if further inspections or maintenance are needed [8]. SHM has the ability to replace the conventional schedule-based inspection and maintenance and save a large amount of time and expenses [9]. However, for the SHM to be applicable in the field as an NDI technique, it must demonstrate 90/95% reliability and probability of detection under operational and environmental conditions of the real structure. 

The fundamental purpose of SHM is to improve the safety of critical systems and reduce maintenance costs [10,11]. Depending on the sensing methodology, SHM techniques can be generally divided into two types: active sensing and passive sensing. For active sensing, both actuators and sensors are required. Actuators emit an excitation on the monitored structures and then it is recorded by sensors. By analyzing the difference between signals, information about the presence and the characteristics of damage can be determined. This method provides more information; meanwhile, it makes the monitoring system more complicated [12]. For passive sensing, only sensors are integrated with the structure and the dynamic response of the structure is recorded constantly. It allows the system to detect possible impacts in real time, although an energy efficient system is required to optimize its power consumption as impact events are random and transitory and the structure is required to be monitored continuously in service [11,13,14].

An impact on a thin plate will create a type of elastic wave, known as Lamb wave, which propagates through the plate. However, Lamb waves are multi-modal and different modes are excited at different frequencies, and it varies with impactor composition, angle of impacts, temperature and other varying parameters [13], which makes impact characterization a real challenge for aircraft structures. Many researchers have developed methodologies for detecting and localizing impact events on aeronautical structures. However, this information alone is not sufficient for passive sensing, as there are many impacts occurring on structures in service, but only the ones that are of high energy and have the potential to cause damage to the structure are of interest. Therefore, reliable impact characterization is of high importance for the assessment and application of any SHM system to real structure. For a real aircraft structure, another challenging factor is the large amount of data collected from sensors during operation, which makes it impossible to be analyzed by conventional numerical algorithms as both time and computational resources are limited [14]. As a result, machine learning methods, particularly neural networks, have been widely applied to extract useful information from large data sets efficiently. Some of the neural networks, like artificial neural networks (ANNs) and convolutional neural networks (CNNs), have been successfully implemented in SHM, especially in damage detection, impact localization and impact classification [15,16,17,18,19,20], as well as the corresponding sensor optimization [21]. 

However, due to the uncertainty contained in both data and model, the reliability of prediction results from neural networks has become a new concern. Conventional neural networks can only give direct prediction results which sometimes could be wrong while Bayesian neural networks (BNNs), an emerging technique in machine learning, allow probabilistic interpretations for predicted outcomes [22]. However, BNNs have not been used for uncertainty quantification of passive sensing systems. Therefore, to quantify the uncertainty (which results from variability in impact scenarios in terms of uncertainty in impact location, impact angle and impact energy) and to improve the reliability and robustness in impact classification, BNNs are applied in this work for the first time. Two types of uncertainty are included in this work, aleatoric and epistemic uncertainties, which is one of the novel contributions. To further compare the BNN performance in terms of computational time and cost, a method called multi-ANN is also utilized in this work. The main objective of this paper is to develop an accurate model to efficiently classify the energy levels of different impact events based on the signals obtained from a network of sensors. The target of the research is to evaluate whether modern probabilistic machine learning algorithms such as BNN will increase the reliability of the prediction, compared to traditional methods such as ANN or CNN. The uncertainty of the developed model is quantified through BNN and the reliability and robustness of the proposed method is tested. To simulate a realistic variability in the impact tests, different impact angles and impactor materials are selected and tested experimentally to generate a large data set. A thorough feature extraction investigation is carried out to find the most suitable parameters to characterize the energy of an impact, which is the most important output from a passive sensing system. 

## 2. Machine Learning Methodologies

The signal processing methods in SHM can be divided into two main categories: model-based methods and data-driven methods [23]. The former focuses on building a mathematical model to reflect the dynamic response to a known impact or damage and it requires sufficient prior knowledge which is not always available in real applications. Some assumptions are made to simplify the process of building such models, the most popular one of which is the linear assumption. It significantly reduces the complexity of models while in fact, non-linearity is quite common in composites and cannot be ignored [24]. Due to this limitation, data-driven methods, especially machine learning, have become the major analytical tool in SHM.

Implementing machine learning techniques in SHM usually contains several steps: data pre-processing, feature extraction and model building. Once features are extracted, they can be fed into an automatic classifier to detect possible anomalies (so-called unsupervised learning) or to predict the energy level and localization of an impact (so-called supervised learning). Unsupervised learning has played an important role in SHM as it does not require any prior knowledge. Rizzo et al. proposed an outlier analysis to detect and quantify cracks in the structure [25]. Ramasso et al. suggested a novel consensus clustering method of acoustic emission time-series to estimate damage sequence in composite [26]. Rastin et al. developed a deep learning-based method to identify and quantify structural damage based on convolutional autoencoders [27].

Compared to unsupervised learning, supervised learning methods show higher performance and wider application prospects when some prior knowledge is available. ANNs, as one of the earliest proposed supervised deep learning methods, are employed for damage detection, impact force reconstruction and impact localization [15,24]. ANNs are a mathematical model which simulates a biological neural system, and hence has the ability to deal with non-linear problems. Compared to normal ANNs, Li et al. proposed a multi-feature fusion method to detect damage based on a support vector machine (SVM) algorithm, which was proved to overperform ANNs [28]. The performance of ANN and SVM for impact detection were compared by Yue et al. [29] to confirm their improved performance. CNNs are a more popular choice as they fuse a complicated feature extractor and classifier into one model, which makes them more attractive for more complex tasks, like damage localization. Abdeljaber et al. first explored a one-dimensional CNNs method used for vibration-based damage detection and localization [30]. A more interesting utilization of CNNs in SHM is to transfer Lamb wave signals to 2D images and it was proved to achieve higher accuracy by Tabian et al. [6]. To solve the problem of missing data in measured signals, a long short-term memory network (LSTM) was suggested by Li et al. [31].

Although the accuracy of conventional machine learning models has reached a relatively high level, more attention should be paid to the reliability of predicting outputs and the robustness of models as structures using SHM are often safety-critical to the whole system, especially aircraft. Jiang et al. proposed a damage detection method based on probabilistic neural networks (PNNs) which provided predicting results with high confidence [32]. However, PNNs require huge computational resources which limit their application in big data and complex models [33]. Huang et al. combined a Bayesian model updating and a vibration-based damage detection method to properly treat the uncertainty in the damage detection process [34]. Morse et al. proposed a strategy for reliably categorizing impacts by their energies based on a combination of ANNs and Bayesian updating method [16]. However, his impact events did not include variability in the angle or material of the impactor.

Since the 1990s, Bayesian theory has been introduced into machine learning to quantify uncertainty in models and datasets, but it was not until 2015 that the efficient framework of BNNs proposed by Blundell et al. made its practical application a reality [35,36,37]. Kendall and Gal presented an improved BNN to quantify aleatoric uncertainty and epistemic uncertainty in computer vision [22]. Yin and Zhu utilized an optimally designed BNN to detect damage in a steel truss bridge [38]. Due to the ability to output probabilistic results fast and accurately, BNNs are chosen as the primary analytical tool for this study. The next section summarizes the fundamentals of BNN before presenting the novel developed methodology. 

## 3. Fundamental of Bayesian Neural Network

### 3.1. Bayesian Neural Network

Bayes theorem is an important theory in the field of statistics, based on which the observable data can be used to infer the probability of event results. The combination of Bayesian theory and machine learning means that uncertainty is contained in conventional machine learning models. Kendall and Gal divided the uncertainty into two categories: aleatoric uncertainty and epistemic uncertainty [22]. The former refers to the uncertainty inside the dataset which could come from many realistic reasons, such as the noise and perturbation of PZT sensors. It cannot be reduced as more data are added into training. However, epistemic uncertainty, also known as model uncertainty, will decrease with the growth of input data into the model. When the model lacks knowledge of some dataset, it is prone to make unreliable decisions based on the datasets trained before. Therefore, epistemic uncertainty directly reflects the reliability of the prediction and can be used to examine the robustness of the model as well.

**Figure 1:**
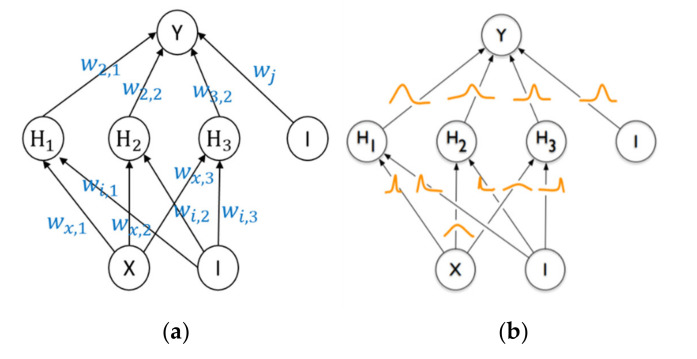
(**a**) ANN where each weight $w_{i,j}$ has a fixed value, (**b**) BNN where each weight is assigned a distribution.

BNNs can evaluate epistemic uncertainty by converting weight and bias parameters of traditional neural networks, like ANNs, from constants to probability distributions (see Figure 1). The following derivations show a probabilistic model and an algorithm called variational inference which was used to implement a practical and efficient BNN [22,37].

#### 3.1.1. Probabilistic Model

A neural network can be regarded as a probabilistic model P(y|x,w) where x represents the input data and w represents the parameters in the network. For classification tasks, y refers to a set of classes and correspondingly P(y|x,w) follows a categorical distribution. Given a dataset with n training points as $D=\left\{x_{i},y_{i}\right\}:$ where $\left|D\right|=n$, it is easy to construct the likelihood function:

$$P\left(D\left|w\right.\right)=\overset{n}{\underset{i=1}{\prod }}P(y_{i}|x_{i},w)\;$$

The maximum likelihood estimate (MLE) of parameters w can be obtained from maximizing this likelihood function and, usually, the negative log likelihood is chosen to optimize objectives, which refers to the cross-entropy of softmax loss for a categorical distribution, shown as:

$$w_{MLE}=\underset{w}{\mathrm{argmax}}\overset{n}{\underset{i}{\sum }}\log P(y_{i}|x_{i},w)$$

Although MLE is widely used in conventional neural network, it is prone to overfitting during training. To overcome the overfitting, regularization can be introduced by multiplying the likelihood with a prior distribution $P\left(w\right):$

$$P\left(w\left|D\right.\right)\propto P\left(D\left|w\right.\right)P\left(w\right)$$

Maximizing $P\left(D\left|w\right.\right)P\left(w\right)$ gives the maximum a posteriori (MAP) estimate of w. The learning objectives for categorical distribution here are the softmax loss plus a regularization term coming from the log prior:

$$w_{MAP}=\underset{w}{\mathrm{argmax}}\overset{n}{\underset{i}{\sum }}\log P\left(y_{i}|x_{i},w\right)+\log P\left(w\right)$$

Both MLE and MAP give point estimates of w, which are not always reliable. Bayesian inference aims to calculate the posterior distribution of the weights based on training data $P\left(w\left|D\right.\right),$ so that the uncertainty parameters can be quantified. 

#### 3.1.2. Variational Inference

Unfortunately, it is impossible to obtain analytical solutions to P(w|D). Therefore, an approximation algorithm is considered. To approximate the posterior distribution, a variational distribution is defined as q(w|θ). The similarity between q(w|θ) and P(w|D) can be measured by the Kullback–Leibler (KL) divergence, which is defined as

$$\mathrm{KL}\left[q\left(w\left|\theta \right.\right)P\left(w\left|D\right.\right)\right]={Ε}_{q\left(w\left|\theta \right.\right)}\log\frac{q\left(w\left|\theta \right.\right)}{P\left(w\left|D\right.\right)}$$

After applying the Bayes theorem to the posterior distribution P(w|D) and making some manipulations, the cost function can be given as:

$$\mathrm{KL}\left[q\left(w\left|\theta \right.\right)P\left(w\left|D\right.\right)\right]=\mathrm{KL}\left[q\left(w\left|\theta \right.\right)P\left(w\right)\right]-{Ε}_{q\left(w\left|\theta \right.\right)}\log P\left(D\left|w\right.\right)+\log P\left(D\right)$$

The first two terms on the right hand side of the equation are also known as the variational free energy $F\left(D,\theta \right).$ In order to minimize the KL divergence between $q\left(w\left|\theta \right.\right)$ and $P\left(w\left|D\right.\right)$, the divergence free energy requires to be minimized:

$$F\left(D,\theta \right)=\mathrm{KL}\left[q\left(w\left|\theta \right.\right)P\left(w\right)\right]-{Ε}_{q\left(w\left|\theta \right.\right)}\log P\left(D\left|w\right.\right)$$

By rearranging the KL term, the above equation can be written as:

(1) $$F\left(D,\theta \right)={Ε}_{q\left(w\left|\theta \right.\right)}\log q\left(w\left|\theta \right.\right)-{Ε}_{q\left(w\left|\theta \right.\right)}\log P\left(w\right)-{Ε}_{q\left(w\left|\theta \right.\right)}\log P\left(D\left|w\right.\right)$$

It is obvious that all three terms in Equation (1) contain the expectation of $q\left(w\left|\theta \right.\right)$. Therefore, by sampling $w^{\left(i\right)}$ from variational distribution $q\left(w\left|\theta \right.\right)$, this expression can be approximated and the final cost function is given by:

(2) $$F\left(D,\theta \right)\approx \frac{1}{M}\overset{M}{\underset{i=1}{\sum }}\left[\log q\left(w^{\left(i\right)}\left|\theta \right.\right)-\log P\left(w^{\left(i\right)}\right)-\log P\left(D\left|w^{\left(i\right)}\right.\right)\right]$$

where M is the batch size. Generally, not all the data are input into the network in one training process (also known as an epoch) due to the inefficiency. Instead, the whole dataset is split into smaller batches and then fed into the network, which enables this algorithm to process large-scale data.

#### 3.1.3. Training and Prediction

A training process in the neural network always includes a forward-propagation and a backward-propagation. For the forward-propagation, a single sample is drawn from the variational posterior and used to calculate the cost function, Equation (2). A popular choice is to assume the variational posterior as a Gaussian distribution and then θ can be represented as $\theta =\left(\mu ,\;\sigma^{2}\right)$ where μ is the mean vector of the distribution and σ is the standard deviation vector. Naturally, the number of parameters in a BNN is doubled compared to a conventional neural network.

During a backward-propagation, μ and σ are updated by calculating their gradients. A method called reparameterization trick is applied here to sample from a given distribution and then map the sampled ϵ to the gradients by using a transfer function $t\;\left(\mu ,\;\sigma ,\;\epsilon \right).$ Taking a standard normal distribution as an example $\epsilon \;\sim \;N\;\left(0,\;1\right)$ and defining $\sigma =\log\left(1+\exp\left(\rho \right)\right)$ to ensure that σ is always non-negative, the deterministic function can be given as:

$$t\left(\mu ,\rho ,\varepsilon \right)=\mu +\log\left(1+\exp\left(\rho \right)\right)\cdot \varepsilon$$

where · represents pointwise multiplication.

To make a prediction based on the well-trained model, BNNs utilize Bayes theorem, according to which, mathematical description of prediction can be given as:

(3) $$P\left(y\left|x,D\right.\right)=\int P\left(y\left|x,w\right.\right)P\left(w\left|D\right.\right)dw$$

where x is a testing data point which the model has not seen before, and u is the unknown label of x. Generally, BNNs can utilize the well-trained model with probabilistic parameters to make a set of predictions. This process is also known as Monte Carlo (MC) sampling or simulation. By using an MC sampling from the well-trained BNN, the predicting results can be counted. Calculating Equation (3) should be equivalent to averaging results from an infinite set of neural networks, which is not possible due to the limitation of computational resources. In this work, a finite set of ANNs, called multi-ANNs, is applied to approach the ideal results and compare it with BNNs.

After obtaining a set of predictions, epistemic uncertainty in the weights is easy to be measured. For a classification task, this can be approximated as follows:

$$P\left(y=c\left|x,X,Y\right.\right)\approx \frac{1}{T}\mathrm{argmax}\left(f^{w}\left(x\right)\right)$$

where c is the label of the class, T is the number of MC sampling and w is a set of masked weights which follow $w\sim q\left(w\left|\theta \right.\right).$ A set of predictions can form a probability vector that can be further summarized to calculate the uncertainty by using the predictive entropy H:

$$H=-\overset{C}{\underset{c=0}{\sum }}P_{c}\log P_{c}$$

Higher predictive entropy corresponds to higher epistemic uncertainty. The predictive entropy directly measures the magnitude of epistemic uncertainty and can be an appropriate metric to reflect the confidence of prediction for each test sample. 

### 3.2. Multi-Artificial Neural Network 

**Figure 2:**
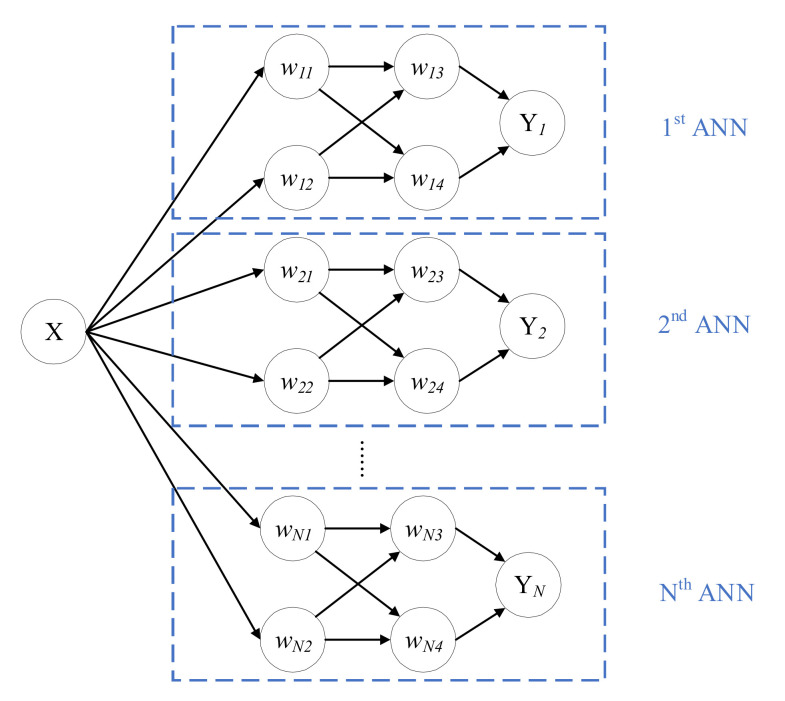
An example of multi-ANNs.

Multi-ANNs are a finite set of conventional ANNs with the same architecture. Figure 2 shows a simple example of an N-ANNs, each of which has two layers and four neurons in total.

Multi-ANNs can be regarded as a type of ensemble learning algorithm, similar to the bootstrap aggregating (also known as bagging) algorithm. The bagging algorithm obtains varied results by drawing samples from the whole training dataset to input into the network several times while in multi-ANNs, probabilistic results are given by inputting the whole dataset into *N*-ANNs whose initial weights and biases follow a specified distribution, usually Gaussian distribution. Based on these *N*-ANNs, the testing dataset is used to make *N* sets of predictions. Considering each single testing sample, *N* predicting results should follow varied unknown categorical distributions, which are similar to those from the BNN. The aim to apply the multi-ANN method in this study mainly consists of two parts: comparing the ability of multi-ANN and BNN to measure the uncertainty in diagnosis and comparing the temporal and computational efficiency of both methods. To achieve this, the parameters and metrics of BNNs and multi-ANNs will be set consistently.

## 4. Data Acquisition and Pre-Processing 

In order to simulate variability of impact scenarios which may occur on real structures, an experimental set up has been designed and impact test rig built, capable of varying impactor height, angle, material and location [39,40]. There are still limitations to how high the impact energy can be; however, as it was demonstrated from the published work, the impact energy response is scalable [41]. Therefore, the proposed impact test campaign is an acceptable case study for the development of the methodology, which can be then extended and upscaled to more complex structures, geometries and impact scenarios in future work. 

### 4.1. Experimental Setup

**Figure 3:**
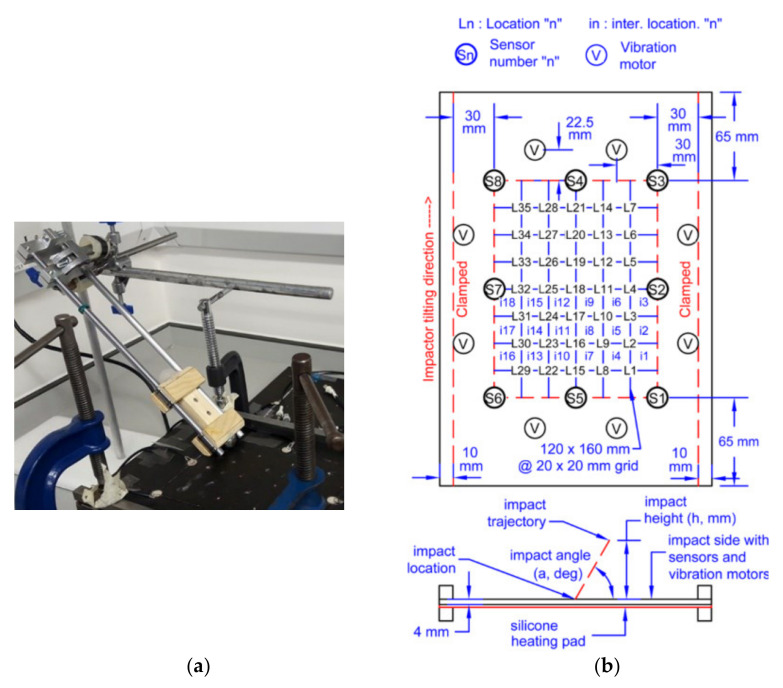
Experimental setup and configuration. (**a**) Impact set up; (**b**) Experimental configuration of the 8 PZT sensors and 35 impact locations.

The setup consisted of a drop impact tower, a fixture for holding the specimen and a 200 mm × 290 mm flat quasi-isotropic panel with the following layup [0/+45/−45/90]_2s_ made of M21 T800s carbon fiber prepregs, as shown as Figure 3a [41]. The flat plate was placed on a silicone heating mat with a temperature control unit to adjust the temperature for different experiments (25 °C as a baseline). Eight vibration motors were placed along the four edges of the plate to simulate background noise/vibration and eight PZT sensors (PIC 255) were bonded to the periphery of the panel. The rectangular area bounded by the sensors was split into a 7 × 5 grid which represents 35 different impact locations, shown in Figure 3b. An 8-channel oscilloscope (NI PXI-5105) was connected to the sensors to record signals with a sampling rate of 2 MHz via 10× attenuation oscilloscope probes. In total, 100,000 samples were obtained from the experimental test campaign. 

Impacts of different energy levels were generated by dropping a 20 mm diameter impactor with different added mass. Impact angle was also varied by tilting the guiding rail. For each location and configuration, each impact was repeated four times. Therefore, a total of 140 tests were contained in each impact case defined in Table 1. Impact energy levels were labelled as 0, 1 and 2 based on the gravitational potential energy which can be calculated from the formula E=mgh, where E is the potential energy and h is the drop height. Case A was performed to build a baseline model and measure the uncertainty for impacts under the baseline temperature (25 °C) and angle (90°). Case B was used to verify the reliability and robustness of the trained model when impacts under different angles were tested.

### 4.2. Data Pre-Processing

If the data obtained from the PZT sensors cannot contain high levels of noise and too much information (e.g., if the discreet signals are used in their entirety), it could result in wrong regularization and prediction. Therefore, an appropriate pre-processing step is necessary which mainly includes two steps: noise reduction and feature extraction, which are presented next.

#### 4.2.1. Noise Reduction

**Figure 4:**
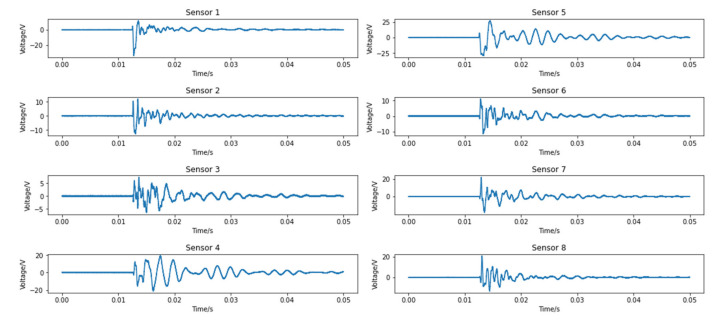
Sample signals recorded by 8 PZT sensors from impact at location 1.

The raw data from the PZT sensors for each impact were stored in a matrix consisting of 100,000 rows and 8 columns. Each column represented a sensor, and each sensor was sampled 100,000 times continuously. Figure 4 shows an example of the signals from eight PZT sensors under a specific impact at location 1. Before the impact occurred, there was a long silent period during which the voltage did not change, and this period could be ignored as it did not make any contribution.

**Figure 5:**
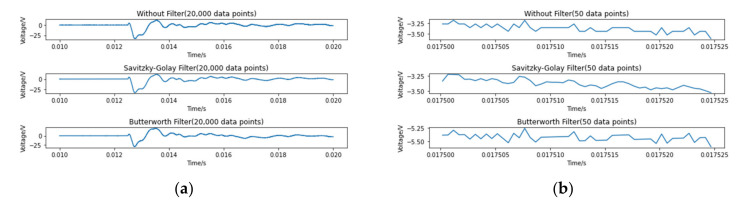
Sample signal with different filters: (**a**) 20,000 data points (**b**) 50 data points.

Although the signals appear to be smooth in Figure 4, there are a large number of micro-fluctuations inside the signals when the image is magnified. These fluctuations come from the noise inside the apparatuses, which can be problematic for further data manipulation. Therefore, applying filters in data processing is necessary. Two filters, a Savitzky-Golay filter (SGF) and a Butterworth high-pass filter, are applied here independently to determine which one performs better. The SGF is a popular 1-D filter that utilizes polynomial to achieve the least square fitting in a sliding window. An SGF with the window length of five and the order of the polynomial two is used after some adjustments to parameters. Separately, a third order Butterworth high-pass filter with a cut-off frequency of 100 Hz is also applied to compare which filer is most appropriate. Figure 5a shows the effect of filters on the main part of the signal and Figure 5b shows a close-up. It is obvious that the SGF significantly smooths the signal and outperforms the Butterworth filter, without losing important information contained in the signal. Finally, this filter was applied to the data from each case.

#### 4.2.2. Feature Extraction

**Figure 6:**
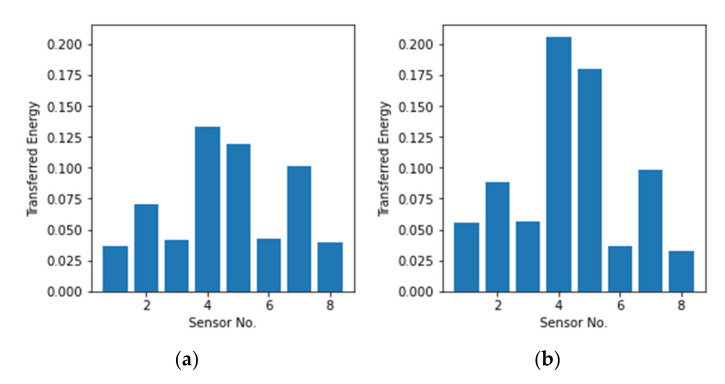
Transferred energy plots for (**a**) Case A1 and (**b**) Case A2 impacts.

The dataset after noise reduction still cannot be used as input to the neural network directly as it is too large. Therefore, feature extraction is necessary. Feature extraction mainly consists of two categories: physical and non-physical features. To obtain a well-fitting non-linear model, physical features play a more important role. Considering the efficiency, simple features that are easy to extract are chosen to classify the energy level of different types of impacts. A common feature used in SHM to classify the energy contained in a signal is its peak amplitude as it reflects the peak value of energy absorbed by the material during the impact. However, this parameter does not contain any information on the duration of the impact which is directly related to the mass (small mass, large mass), or the material (hard, soft) of the impactor, which are necessary for a detailed impact categorization. Compared to the peak amplitude, a more direct mapping between the energy and the signal is obtaining the transferred energy of the impact which is the integral of the absolute values of the voltage signal. Figure 6 shows the transferred energy of two impacts at the same location, respectively from case A1 and case A2. The mass of the impactor in case A2 is twice as large as that in case A1 so that the transferred energy of case A1 should be doubled as well, which corresponds to the results. 

**Figure 7:**
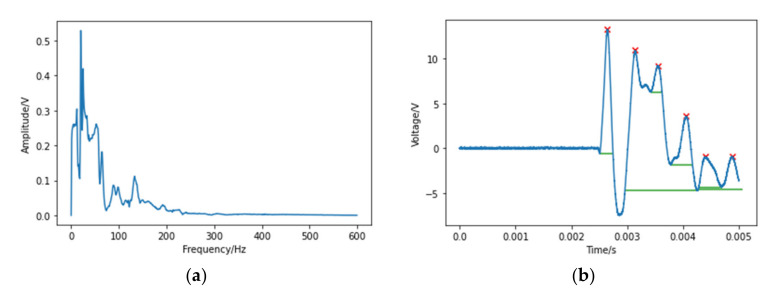
(**a**) FFT of the filtered signal, (**b**) main peaks and the time intervals of a signal.

Transferred energy is an appropriate feature for perpendicular impacts, while for angled impacts it may not give convincing results as some of the energy is dissipated through sliding or friction. To solve this problem, some other features are considered and tested in this work. The frequency components corresponding to the maximum amplitude are extracted from signals by a fast Fourier transform (FFT) method. FFT is a more efficient algorithm obtained by improving the algorithm of discrete Fourier transform (DFT). It converts the time-domain signal into the frequency-domain signal, so as to extract the required frequency-domain features. Figure 7a shows an FFT example of the filtered signal that can be found in Figure 5. The frequency of the maximum amplitude is 20 Hz. 

Wavelength is not a common feature for energy classification as it does not directly provide strength information contained in impact events. However, it can be combined with the transferred energy to train the neural networks in some special impact cases that do not cause significant amplitude but still contain enough energy to cause damage. The time interval of the main peak can build a mapping into the wavelength of the time-domain signal. Therefore, by finding the time interval of the largest peak, the wavelength can be measured. Figure 7b indicates the main peaks by red “×” and their time intervals by green lines.

The features are extracted from each senor for every impact and Table 2 shows an example of features extracted from case A. It is obvious that the difference between magnitudes of these three features is significant, which is easy to lead to non-convergence of the networks. Therefore, these features require to be scaled into a similar magnitude. A standard scaler is applied here to make the processed data follow the standard normal distribution with the mean value as 0 and the standard deviation as 1. After scaling, the features can be fed into the neural networks according to different experimental requirements. In the result presented in the next section, the first feature, transferred energy, will be first applied to perpendicular impact data, including cases A and B. Then the second and third features will be applied to both perpendicular and angled impact data to evaluate whether they provide additional information for those impacts.

## 5. Results and Discussion

**Figure 8:**
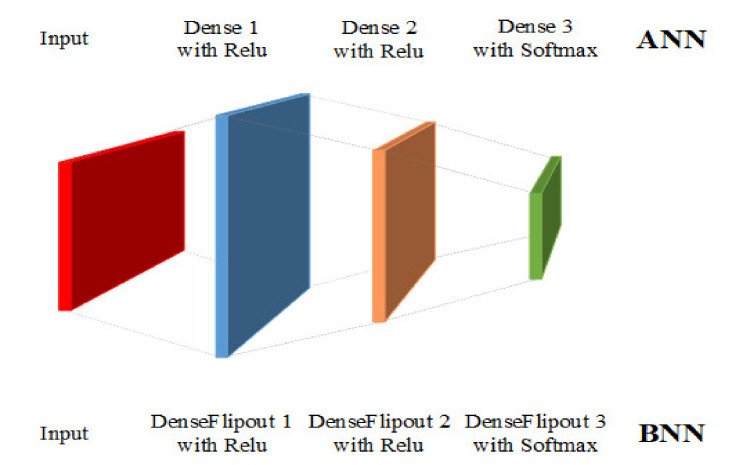
The structure of the BNN and ANN used in this study.

This section introduces the results obtained from both BNN and multi-ANNs. The BNN and ANN used in this work have the same architecture, as shown in Figure 8, and are implemented by TensorFlow and TensorFlow Probability. The input layer includes 16 neurons or 8 neurons depending on which features are used. The second and third layers contain 64 and 32 neurons, respectively. For the output layer, it has three neurons as the number of classes is three. A DenseFlipout layer from TensorFlow Probability is used in BNN to achieve the variational inference method mentioned in Section 3.1, and the default prior distribution of the kernel follows a standard normal distribution. These two networks are first trained by the data from case A to obtain a baseline model and then tested by the other data from case A to examine the performance of the baseline model. The purpose of training a baseline model is to show the ability of the proposed method to classify the impacts correctly and the reliability of the BNN when it makes predictions based on a dataset which it has never seen. Therefore, this baseline model will be further tested by data in case B to demonstrate the influence of uncertainty from impacts in different angles.

### 5.1. Perpendicular Impacts 

#### 5.1.1. Single ANN

**Figure 9:**
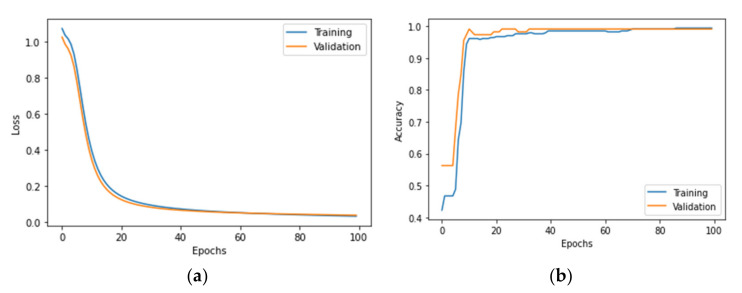
The plots of (**a**) loss and (**b**) accuracy for a single ANN.

For impacts perpendicular to the plate surface, the transferred energy is an appropriate feature to verify the performance of the proposed models. The overall number of samples from case A is 560. These data are first randomly split into training, validation, and testing datasets in 6:2:2 ratio to adjust and optimize the hyperparameters of the network. These hyperparameters include the number of neurons, activation function, learning rate, batch size and epochs. To fit the model and evaluate the performance of training, validation and testing, the categorical cross-entropy is used as the loss function and accuracy is applied as an appropriate metric. An Adam optimizer with a learning rate of 0.001 is adopted to achieve a fast and efficient fitting and the number of epochs is set as 100 to reach a sufficient convergence. These data are firstly input into a single ANN and the plots of loss and accuracy are shown in Figure 9. The decline of the loss curve is very smooth, which proves that the model fits well. The accuracy curve has a satisfying shape as well and the accuracy of evaluation reaches 99.1%. The results show that ANN can classify the energy level of impacts with high accuracy based on the transferred energy.

#### 5.1.2. Bayesian Neural Network 

**Figure 10:**
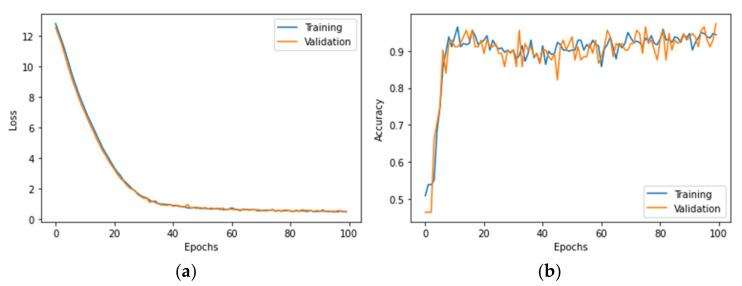
The plots of (**a**) loss and (**b**) accuracy for the BNN.

The BNN accepts similar hyperparameters to the ANN and the only difference between them is that the former uses the Flipout estimator with a well-designed KL divergence function to optimize the probabilistic parameters and the learning rate is set as 0.01. The same data are input into the proposed BNN and the plots of loss and accuracy are shown in Figure 10. The shape of the loss curve is similar to Figure 9a, while the accuracy curve is quite different from Figure 9b. The accuracy first rises rapidly to about 0.9 and then fluctuates significantly as the training progresses. The fluctuation mainly comes from the probabilistic parameters in the BNN, for which these parameters are random variables and follow a probability distribution.

**Figure 11:**
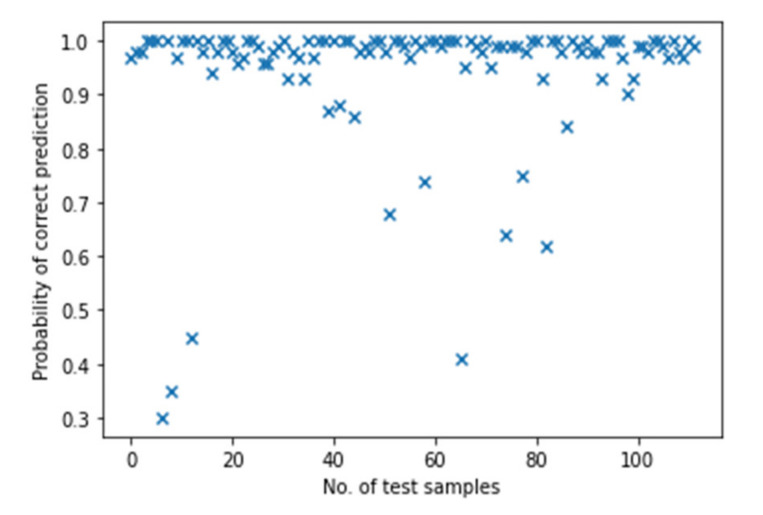
The probabilistic results from Monte Carlo sampling.

For a more in-depth evaluation, the accuracy of BNN reaches 94% max, which is lower than the result from the single ANN. However, the decline of evaluation accuracy is accompanied by the ability to measure the uncertainty and the improvement of the reliability of the BNN. A simple way to show this is to make several predictions based on the same trained model and count the probability of the correct predictions, which is also known as the Monte Carlo method. Figure 11 shows the results obtained from 100 Monte Carlo sampling. It counts the probability of correct predictions (P) over the total 100 predictions for 112 test samples and the results show that for most of the test samples, *P* reaches a satisfying level.

Since the impact categorization is based on detecting the impact energies in different classes, it is important to know how close the predicted impact energy levels are to the true values. This is important when the high impact energies fall close to the damage initiation levels and a wrong prediction can cause a significant cost to the performance of the structure. Therefore, a new measure related to the reliability and confidence of the decision making is introduced here. In order to distinguish different levels of predictions and intuitively reflect their reliability, the predictions are further classified as follows:

- 0.9<P≤1 **Reliable**
- 0.1<P≤0.9 **Unreliable**
- 0<P≤0.1 **False**

**Figure 12:**
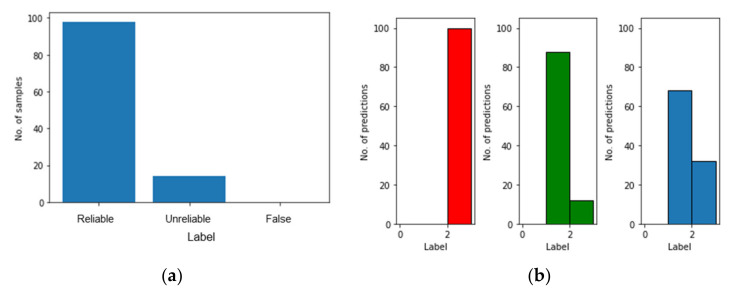
The distribution of classified results with BNN. (**a**) Single test sample. (**b**) Three single testing samples.

The distribution of classified results is shown in Figure 12a. Most predictions are “reliable” while there are still a few “unreliable” results. Recalling that the accuracy of evaluation is 94%, the percentage of “reliable” predictions is lower than this, reaching around 88%. This is because the accuracy of evaluation comes from a single prediction while Monte Carlo sampling provides more information about the reliability of results which influences the percentage of “reliable” predictions. By adjusting the threshold of P, this percentage varies as well. Furthermore, for a single testing sample, Monte Carlo method could obtain a categorical distribution which the predictions should follow.

Figure 12b shows the results of a test case where three testing samples were selected and their categorical distributions of predictions are presented. It is clear that from left to right, the uncertainty of predictions and the probability of false prediction gradually increase. In order to measure the epistemic uncertainty of these test samples, the predictive entropy presented in Section 3.1.3 is calculated as 0.05, 0.48 and 0.66, respectively. It also proves the phenomenon that the higher predictive entropy should correspond to the higher probability of false prediction.

#### 5.1.3. Multi-ANN 

**Figure 13:**
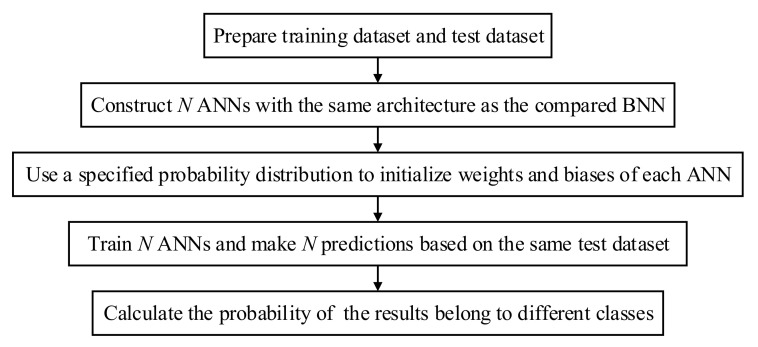
A flowchart of building multi-ANNs.

From Section 3.1, the results from the BNN should be equivalent to averaging results from an infinite set of ANNs, which is not possible due to the limitation of computational resources. In this section, the number of ANN in the Multi-ANN is set the same as the number of Monte Carlo sampling used in the BNN so that the results of predictions can be compared under the same standard. Multi-ANNs are a finite set of conventional ANNs with the same architecture. The flowchart of how to build multi-ANNs is shown in Figure 13. 

**Figure 14:**
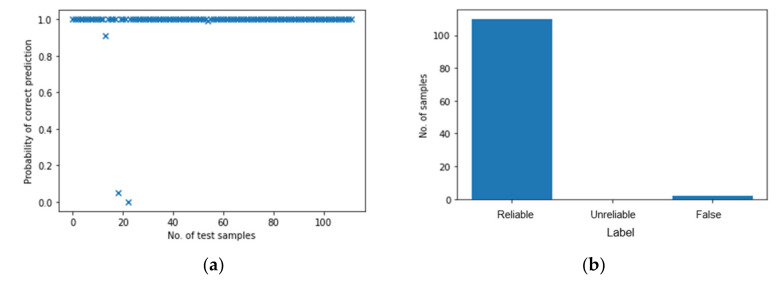

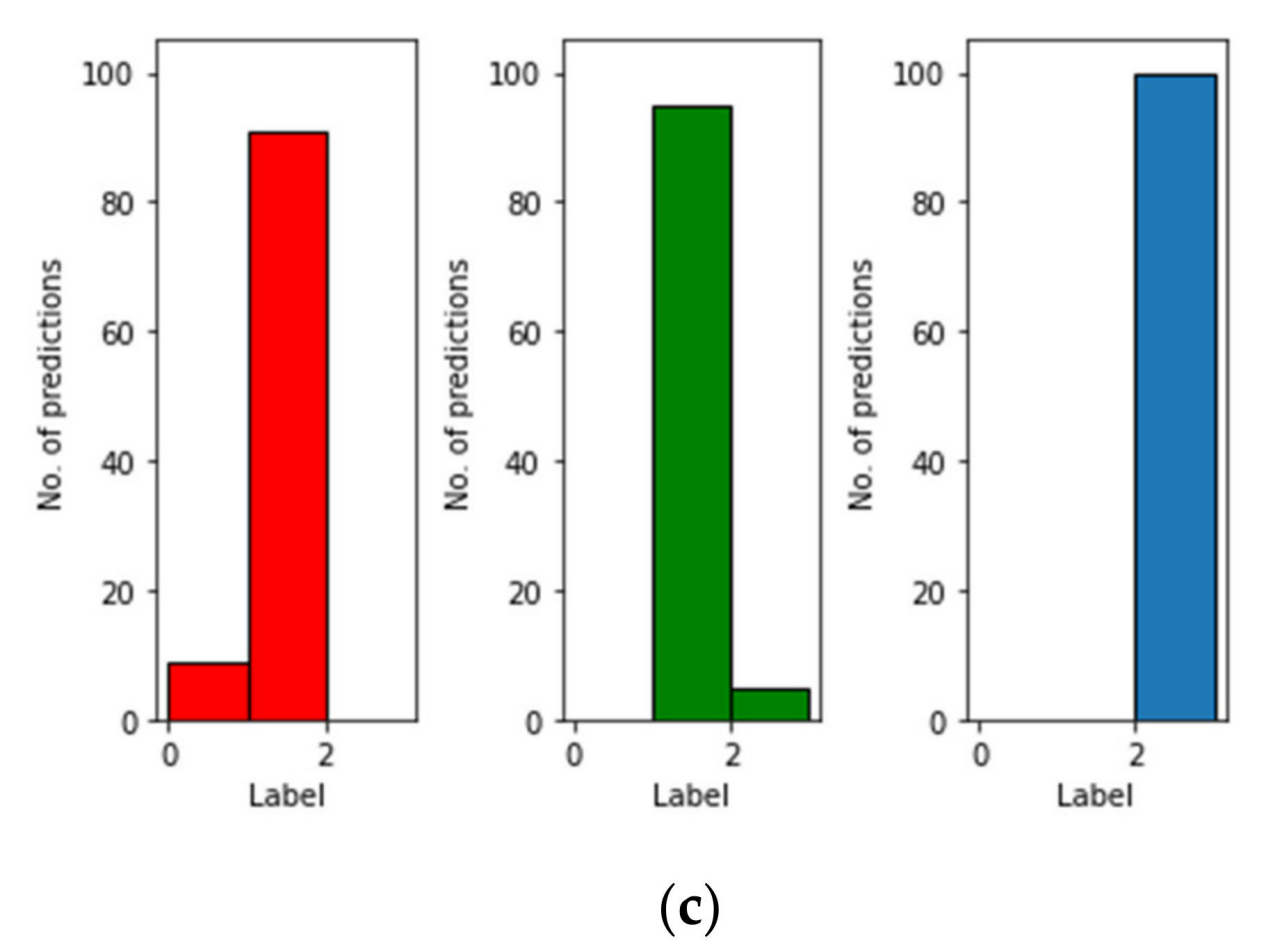
The probabilistic results from 100 ANN. (**a**) Probability of correct predictions. (**b**) Their distribution. (**c**) Results from three test samples.

The probabilistic results from 100 ANN and their distribution are shown in Figure 14. Compared with the BNN (Figure 12), it seems that multi-ANN gives a more reliable result as the “reliable” predictions account for 98% of the total predictions; however, 2% is “false” prediction. In contract, while for the BNN the probability of correct prediction, P, is 95% as “reliable”, there are only 5% “unreliable” predictions and no “False” predictions, which is more important for the application of SHM.

**Figure 15:**
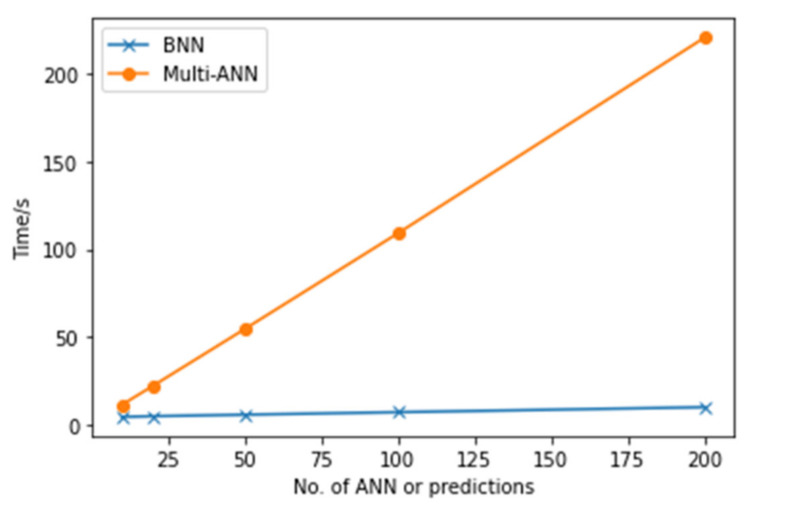
The time efficiency of different numbers of ANNs and Monte Carlo sampling.

**Figure 16:**
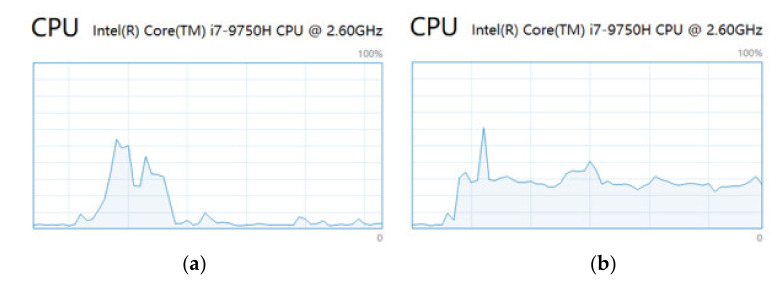
The CPU usage of (**a**) the BNN and (**b**) multi-ANNs.

Although the results from multi-ANN look better than those from the BNN, the efficiency of these two methods also needs considering. To compare the temporal efficiency, the time used by different numbers of ANNs and Monte Carlo sampling is recorded and presented in Figure 15. The time cost of multi-ANN increases linearly and rapidly as the number of ANN increases, while the time cost of the BNN remains stable. Figure 16 shows the computational resource usage of the 100 ANN and of the BNN with 100 times Monte Carlo sampling. The peak of CPU usage is not very different between the two figures, while the multi-ANN cost more computational resources in the same time interval and consume computational resources continuously.

To summarize, both BNN and multi-ANN are able to classify the energy level of perpendicular impacts to an acceptable accuracy. Using the same structure of neural networks, multi-ANNs obtain a higher probability of correct predictions for the test samples compared to BNN, while the probability of false alarm is also higher since BNN has higher probability of unreliable detection (uncertain decision making) but 0% of False classification. However, multi-ANNs require much more time and computational resources than ANNs, which could be the principal limit of applying multi-ANN in the real-life environment. The question now is, which one has better prediction when there is larger variability in the impact scenarios, for example, including angled impact which introduces uncertainty. This will demonstrate which network can better reach a generalization and will be investigated next. 

### 5.2. Angled Impacts 

The previous section used only the data from case A in Table 1 where the impacts are perpendicular to the plate. As expected, both BNN and multi-ANN obtain satisfying results of predictions. The following section assesses the scalability and reliability of the predictive model for classification of impact scenarios that have not been used in the training of the meta-models before and have some certain degree of uncertainty and variability, as these will be more representative of the real operational and environmental conditions. The BNN is first trained with the data from case A and tested with data from both cases A and B. Case A includes 560 samples in total and case B includes 280 samples in total, and 80% of the data from case A are randomly chosen to train the BNN. The other 20% of data from case A, including 112 samples and the same amount of data from case B, are used to test the trained BNN. To compare the effects of different features, the transferred energy is first used as the single input parameter in both BNN and multi-ANN, followed by including multiple features such as the frequency and the time interval of the largest peak, respectively.

#### 5.2.1. Single Feature

**Figure 17:**
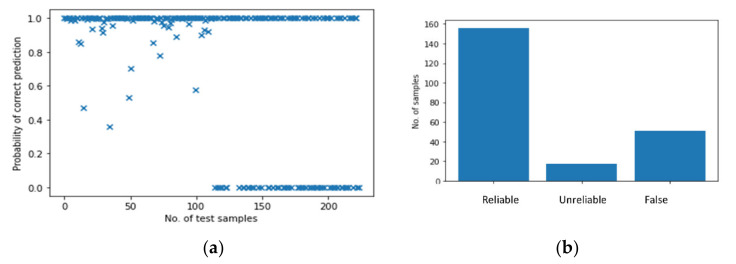
The results of the BNN based on mixed data with transferred energy. (**a**) Probability of correct predictions. (**b**) Their distribution.

The results of the BNN based on mixed data which are only trained and tested with a single feature, i.e., transferred energy, are shown in Figure 17. In Figure 17a, a total of 224 samples are used for testing, of which the first half are from case A and the second half are from case B. The predictions of the first half are more reliable than those of the second half as there are many test samples with very low probability of correct prediction *P*. Figure 17b highlights this by demonstrating the number of “False” predictions which reaches 51, which is 22% of overall test cases (A+B), but accounts for 45% of the samples from case B. In other words, the accuracy of predicting case B (which has not been used at all in the development and training of the neural network) is only 55% while the accuracy of case A still remains at a high level, which is to be expected. By investigating the results in depth, it is found that all the “False” predictions are from case B2, which are impacts of 50 mm height and contain an energy of 49 mJ. This result is not surprising as the characteristics of perpendicular impacts and angled impacts are very different, hence a model trained by perpendicular impacts cannot predict angled impacts with high probability. The summary of the overall results in presented in Table 3. 

**Figure 18:**
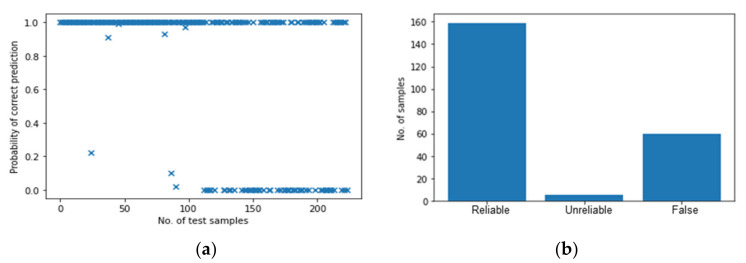
The results of multi-ANN based on mixed data with transferred energy. (**a**) Probability of correct predictions. (**b**) Their distribution.

To compare the scalability between BNN and multi-ANN, the multi-ANNs are trained in the same way and their results are presented in Figure 18. The first half of the test samples show the same trend as that in Section 5.2.1 as they all belong to perpendicular impacts. For the second half, the multi-ANNs have an increased “False” prediction as the number of “False” predictions reaches 61, accounting for 55% of the samples from case B. Therefore, the multi-ANNs perform worse than the BNN when the well-trained models are tested by the data they have never seen. Moreover, considering the huge gap in the temporal and computational efficiency between the two models, the BNN is undoubtedly a better choice.

Generally, the transferred energy feature alone is not an appropriate feature for the angled impacts because the probability of wrong predictions is nearly 50%. Therefore, to improve the accuracy of predictions, the other two features mentioned in Section 4.2.2, namely the signal frequency at maximum amplitude and the time interval of the largest peak, are respectively combined with the transferred energy to train and test the model.

#### 5.2.2. Multiple Features

**Figure 19:**
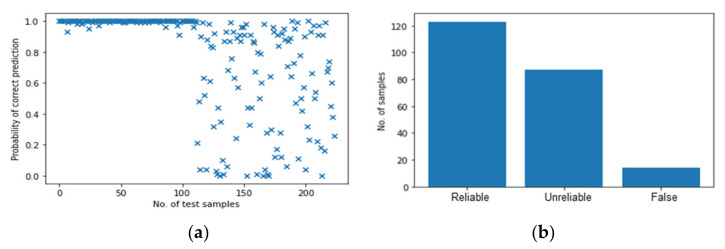
The results of BNN with transferred energy and frequency. (**a**) Probability of correct predictions. (**b**) Their distribution.

In this section, the transferred energy combined with two other features are used to develop and test the BNN only, as the multi-ANNs have demonstrated not to have higher performance in detecting angled impacts. Transferred energy combined with the frequency is firstly used as input to the BNN. Figure 19 shows the results of the BNN based on mixed data with multiple input features including the transferred energy and the frequency components corresponding to the maximum amplitude. Compared to Figure 17a, the predictions for perpendicular impacts in Figure 19a become more accurate and reliable as the probability of correct identification, *P*, for the first half of the test samples is mainly around 1.0. However, for the second half of the tests, Figure 19a shows worse results and the model cannot predict the angled impacts correctly as the scatter is very high. Therefore, the choice of frequency content as an additional input feature is not suitable for angled impacts. 

**Figure 20:**
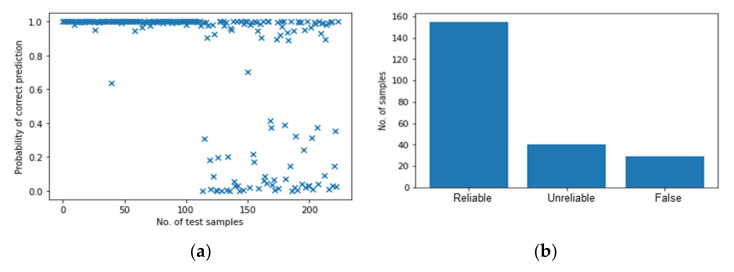
The results of BNN with transferred energy and time interval. (**a**) Probability of correct predictions. (**b**) Their distribution.

Considering multiple features of transferred energy and the time interval, Figure 20 shows the results of the BNN based on mixed data with mixed features. Similarly, compared to Figure 17a, the predictions of perpendicular impacts in Figure 20a are more accurate and reliable. For the part of angled impacts (i.e., the second half of the test samples), it is clear that the mixed features increase the probability of correct predictions, and the number of “False” predictions decreased. It demonstrates that the additional feature, time interval, helps to identify the angled impacts although the accuracy is still not as good as expected.

To conclude, as an additional feature, the frequency at maximum amplitude is not an appropriate feature for classifying the angled impacts as the predictions are completely random. In comparison, the time interval of the largest peak helps to identify both the perpendicular and angled impacts. With the number of “reliable” predictions remaining the same, it reduces the number of “False” predictions. These results emphasize the importance of including variability of the impact scenarios in the development and testing stage. Meanwhile, both multi-ANN and BNN show the potential to output the confidence and probability of predictions for data that have never been seen before. However, considering the efficiency, BNN significantly reduces the time and CPU usage, which makes it possible to apply BNN in real-life for safety-critical structures. 

In conclusion, the results of both networks are compared in Table 3 for different training sets and different input features. Please bear in mind that the probability of classification is presented for the full testing dataset (case A+B). However, as is demonstrated in Figure 17, Figure 18, Figure 19 and Figure 20, most of the errors are for the second half of the test samples which corresponds to Case B and, for the final conclusion, the probability of classification for classes A and B should be considered separately to comment on the regularization capability of each meta-model, which is the important factor for upscaling of the proposed method to real structures. This point is emphasized in the conclusion section. 

## 6. Conclusions

In this paper, a novel machine learning method for classifying impact energy levels and quantifying the uncertainty based on passive sensing in a flat composite plate is proposed, implemented and evaluated. The proposed method, BNN, is compared with multi-ANN to critically assess the capabilities of both neural networks’ performance, including the accuracy of predictions, the time efficiency and computational resources. The focus of the performance analysis has been on upscaling of such developed machine learning techniques to real structures, where there will be a large variability in the data from the trained ones. For example, what will be the reliability of a meta-model that was developed and tested at a small scale with perpendicular impacts only, in correctly categorizing inclined impacts. The findings of the research study are mainly listed below:

- Both the BNN and single ANN can classify energy levels of perpendicular impacts with high accuracy using the feature of transferred energy, although the accuracy of the single ANN is higher than that of the BNN.
- Both the BNN and multi-ANN can quantify the uncertainty in the mode and calculate the confidence of predicted outcomes. For perpendicular impacts, the confidence of predicted outcomes in the multi-ANN is higher than that in the BNN, while the time and computational resource cost of the multi-ANN are significantly larger than those of the BNN.
- The time and computational resource cost of the multi-ANN increase linearly as the number of ANN used increases, while the cost of the BNN remains stable as the number of Monte Carlo sampling increases. For 100 ANN and 100 times Monte Carlo sampling, the cost of the multi-ANN is significantly larger than that of the BNN.
- For angled impacts, both the BNN and multi-ANN can only reach the accuracy of nearly 50% with the feature of transferred energy, while the multi-ANN tends to make more “False” predictions.
- The dynamics response that the perpendicular and inclined impacts generate in the plate are very different, even if they are of the same mass and height; therefore, if a metamodel is developed for perfect impact scenarios in the laboratory condition (i.e., perpendicular impacts), it cannot predict inclined impacts with high accuracy. It is observed that including other features that can directly relate to the characteristic response that each impact scenario generates in the structure, can improve the results, but this needs to be further investigated, including more variability in the impact scenarios. This conclusion also follows the findings in [22] where two step classification is proposed. Therefore, future work will investigate the variability not only of impact angle but also impactor material, mass and size to represent a more realistic variability.
- The mixed features of transferred energy and frequency at maximum amplitude cannot be used to predict energy levels of angled impacts as the results are totally random. The mixed features of transferred energy and time interval of the largest peak show a potential to help to identify both the angled and perpendicular impacts as the number of “False” predictions for angled impacts and “Unreliable” predictions for perpendicular impacts decreases meanwhile.

In summary, both the BNN and multi-ANN can classify the energy levels and quantify the uncertainty for the impact scenarios that they were trained with, i.e., perpendicular impacts, with reasonable accuracy. For angled impacts, the accuracy of classification is not satisfying and some new features which represent the dynamic response of the plate to inclined impacts should be included and researched in future studies. However, due to the ability to quantify the uncertainty, the BNN and multi-ANN can provide additional information that can be further utilized for decision making, in the application of SHM to real structures. Considering the huge gap in the temporal and the computational efficiency between these two methods, the BNN is proved to outperform the multi-ANN and has very broad application prospects in the field of SHM, especially for those safety-critical components and structures. 

The results presented in this paper are a first step in critically assessing the proposed BNN with limited test scenarios. It should be further tested on larger and more complex structures such as stiffened panels and should also include more variability in the impact scenario, to have a more realistic representation of in service loads and impact events. 

## Figures and Tables

**Table 1:** Impact test scenarios.

Case	Material	Height/mm	Mass/g	Angle/°	Temperature/°C	Energy/mJ
A1	Steel	50	100	90	25	49
A2	Steel	50	200	90	25	98
A3	Steel	100	100	90	25	98
A4	Steel	100	200	90	25	196
B1	Steel	50	100	45	25	49
B2	Steel	100	100	45	25	98

**Table 2:** Features extracted from case A. $S_{i}$ represents the sensor number i.

Label	Energy/J(S1) …	Energy/J(S8)	Frequency/Hz(S1) …	Frequency/Hz(S8)	Time int/s(S1) …	Time int/s(S8)
0	0.04968	0.04552	20	58	0.01287	0.00788
0	0.04943	0.04592	20	58	0.01293	0.00806
0	0.04958	0.04609	20	58	0.01302	0.00791
0	0.04911	0.04601	20	58	0.01298	0.00794
0	0.04062	0.04641	20	20	0.03223	0.01758
…	…	…	…	…	…	…
1	0.07705	0.03190	21	21	0.02126	0.01660
…	…	…	…	…	…	…
2	0.10343	0.05218	21	20	0.02105	0.01538

**Table 3:** Comparison of BNN performance to ANN.

Meta Model	Input Feature *	Training Set	Testing Set	Reliable Classification	Unreliable Classification	False Classification
BNN	1	A	A	94%	6%	0%
BNN	1	A	A+B	70% (only 55% for B)	8%	22%
BNN	1+2	A	A+B	70%	8%	22%
BNN	1+3	A	A+B	54%	39%	7%
Multi-ANN	1	A	A	98%	0%	2%
Multi-ANN	1	A	A+B	70% (only 44% for B)	2%	28%
Multi-ANN	1+2	A	A+B	70%	3%	27%
Multi-ANN	1+3	A	A+B	70%	18%	12%

* Input features: 1. Transferred energy, 2. Signal frequency at maximum amplitude and the time interval of the largest peak.
